# Antibody Directed against Human YKL-40 Increases Tumor Volume in a Human Melanoma Xenograft Model in Scid Mice

**DOI:** 10.1371/journal.pone.0095822

**Published:** 2014-04-21

**Authors:** Johannes Salamon, Tatjana Hoffmann, Eva Elies, Kersten Peldschus, Julia S. Johansen, Georg Lüers, Udo Schumacher, Daniel Wicklein

**Affiliations:** 1 Institute of Anatomy and Experimental Morphology, University Cancer Center, University Medical Center Hamburg-Eppendorf, Hamburg, Germany; 2 Department of Radiology, University Cancer Center, University Medical Center Hamburg-Eppendorf, Hamburg, Germany; 3 Department of Interdisciplinary Endoscopy, University Cancer Center, University Medical Center Hamburg-Eppendorf, Hamburg, Germany; 4 Oncology and Medicine, Herlev Hospital, Copenhagen University Hospital, University of Copenhagen, Copenhagen, Denmark; University of Torino, Italy

## Abstract

Induced overexpression of the secretory protein YKL-40 promotes tumor growth in xenograft experiments. We investigated if targeting YKL-40 with a monoclonal antibody could inhibit tumor growth. YKL-40 expressing human melanoma cells (LOX) were injected subcutenously in Balb/c scid mice. Animals were treated with intraperitoneal injections of anti-YKL-40, isoptype control or PBS. Non-YKL-40 expressing human pancreatic carcinoma cell line PaCa 5061 served as additional control. MR imaging was used for evaluation of tumor growth. Two days after the first injections of anti-YKL-40, tumor volume had increased significantly compared with controls, whereas no effects were observed for control tumors from PaCa 5061 cells lacking YKL-40 expression. After 18 days, mean tumor size of the mice receiving repeated anti-YKL-40 injections was 1.82 g, >4 times higher than mean tumor size of the controls (0.42 g). The effect of anti-YKL-40 on the increase of tumor volume started within hours after injection and was dose dependent. Intratumoral hemorrhage was observed in the treated animals. The strong effect on tumor size indicates important roles for YKL-40 in melanoma growth and argues for a careful evaluation of antibody therapy directed against YKL-40.

## Introduction

The secretory glycoprotein YKL-40, also designated human cartilage glycoprotein-39 (HC-gp39) [1], 38-kDa heparin-binding glycoprotein (gp38k) [2], chitinase-3-like-1 (CHI3L1) [3], and chondrex [4], belongs to the mammalian chitinase like family and binds collagen- [5], heparin-, hyaluronan- [6] and chitin, but has no chitinase activity [1].

YKL-40 is mainly produced by macrophages [3], [7], neutrophils [8] and cancer cells [9]. YKL-40 plays a role in cell proliferation and differentiation [10]–[12], angiogenesis [13]–[17], inflammation [18]–[21], remodeling of the extracellular matrix [22] and protects against apoptosis [23]. A receptor for YKL-40 has not been identified, yet.

Plasma levels of YKL-40 are elevated, compared to healthy subjects, in patients with different types of cancer, including melanoma [24], [25] and pancreatic carcinoma [26], [27], and is related to stage (highest levels in metastatic disease) and prognosis [9]. Additionally, application of an anti-YKL-40 monoclonal antibody significantly reduced tumor growth in a human glioblastoma (U87) xenograft model in mice [28].

The aim of the present study was to evaluate the effect of an anti-YKL-40 monoclonal antibody on tumor growth and morphology in a xenograft model of human melanoma and pancreatic adenocarcinoma in scid mice previously established in our lab [29], [30].

## Materials and Methods

### Antibodies

A mouse anti human YKL-40 monoclonal antibody (IgG2bκ) was used in all following experiments. The antibody was raised against human YKL-40 purified from serum-free, conditioned medium from monolayer cultures of the YKL-40 producing human osteosarcoma cell line MG63 and subsequently purified at high-pressure liquid chromatography. Corresponding isotype (IgG2b) mouse antibody was obtained from eBioscience (San Diego, California, USA).

### Cells and Cell Culture

The human melanoma cell line LOX, originally established from a metastatic lymph node [31], and the human glioblastoma cell line U87 were cultured in RPMI 1640 medium, supplemented with 10% fetal bovine serum (FBS), 2 mM L-glutamine, 100 Uml^-1^ penicillin and 100 µgml^-1^ streptomycin (all Invitrogen, Karlsruhe, Germany). Human pancreatic adenocarcinoma cell line PaCa 5061 was established from a primary tumor. Cell culture conditions and a detailed characterization of this cell line were published previously [29]. Human umbilical vein endothelial cells (HUVEC, Promocell, Heidelberg, Germany) were cultivated in ECM (Endothelial Cell Medium, Sciencell, Carlsbad, CA, USA). For injection into the animals, cells were detached from the flask surface using enzyme-free Cell Dissociation Buffer (Invitrogen), washed with PBS and checked for viability. Afterwards, the cells were re-suspended at a final concentration of 5×10^6^ viable cells per ml in RPMI 1640 without supplements.

For proliferation studies, 7.5×10^3^ LOX or U87 cells were seeded in 96-well flat bottom plates (six wells for each different condition) and incubated for 48 h in standard medium with 1 µg/ml recombinant YKL-40 (Quidel, San Diego, CA, USA) or with 0.01, 0.1, 1, 10 µg/ml anti-YKL-40 or with both YKL-40 and anti YKL-40, respectively. Proliferation rate was determined with the XTT based Cell Proliferation Kit II (Roche Diagnostics, Mannheim, Germany) according to the manufacturer’s instructions and compared with unstimulated cells as control.

For tube formation assays, U87 cells were seeded in RPMI 1640 (with supplements) in 6-well flat bottom plates and cultivated for 48 h. Afterwards the medium was removed and replaced by 1 ml ECM supplemented with 0, 5 or 10 µg/ml anti-YKL-40 antibody. After 24 h conditioning, the ECM was removed, filtered and used for the tube formation assays.

### Animal Experiments

The methodology for carrying out the animal experiments was consistent with the UKCCR guidelines for the welfare of animals in experimental neoplasia [32]. The experiment was recommended and supervised by the institutional animal welfare officer, and approved by the local licensing authority (Behörde für Soziales, Familie, Gesundheit, Verbraucherschutz; Amt für Gesundheit und Verbraucherschutz; Billstr. 80, D-20539 Hamburg, Germany) under the project No. 67/07.

All animals used were pathogen-free Balb/c severe combined immunodeficient scid mice aged 9–14 weeks with a weight of 25–30 g at the beginning of the experiments. The mice were housed in filter-top cages and provided food and water ad libitum and their condition was monitored daily. Apart from primary tumor size or ulceration, the general condition of the animals was evaluated by a standardized in house scoring system based on movement/behavior, weight development, food and water intake and fur condition. The mice were killed by cervical dislocation after having been anesthetized by intraperitoneal injection of a weight-adapted dose (10 µl/g bodyweight) of a mixture of 1.2 ml Ketamin (Gräub AG, Bern, Switzerland), 0.8 ml Rompun (Bayer AG, Leverkusen, Germany) and 8 ml saline.

#### Study A

40 mice received a subcutaneous injection of 10^6^ LOX cells (200 µl cell suspension) directly below the right scapula. The experiment was ended after the tumor weight for the first mouse in the experiment exceeded 10% the animal’s weight. 20 mice received regular intraperitoneal injections of 200 µg (approximately 6.7 mg per 1 kg body weight) of anti-YKL-40 monoclonal antibody (in 200 µl PBS) and 20 mice received 200 µl of PBS. The experiment was started on a Monday with injection of the cells (see above). After two days, the mice were injected intraperitoneally with the anti-YKL-40 antibody or PBS, and these injections continued 3 days a week (Monday, Wednesday and Friday) until the experiment was terminated at day 18. Each animal received a total of 7 injections.

#### Study B

20 mice received a subcutaneous injection of 10^6^ PaCa 5061 cells (200 µl cell suspension) directly below the right scapula. As above, the mice received injections of 200 µg anti-YKL-40 or mouse IgG2b isotype (10 animals each group). Animals were sacrificed when the tumors ulcerated or reached 10% of the animals’ original weight.

#### Study C

For monitoring tumor growth by MRI, 10 mice received a subcutaneous injection of 10^6^ LOX cells (200 µl cell suspension) into the flank. The first MRI scan was taken after 8 days, followed by intraperitoneal injections of 200 µg anti-YKL-40 monoclonal antibody in 5 mice or 200 µg of mouse IgG2b isotype control in 5 mice (in 200 µl of PBS). Consecutive antibody injections were given every second day. The next MRI images were taken after six hours and then at day 2, day 5 and day 7. The animals were sacrificed 17 days after the injection of cells.

#### Study D

For monitoring early increase in tumor volume and bleeding by MRA, 4 mice received a subcutaneous injection of 10^6^ of LOX cells (200 µl cell suspension) into the flank. The first MRI scan was taken after 8 days, followed by an intravenous injection of 10, 100 or 400 µg of anti-YKL-40 antibody or 400 µg IgG2b isotype control (in 200 µl of PBS, respectively). A second MRI scan was taken six hours after this antibody injection and a third MRI scan after 24 h and the animals were sacrificed directly afterwards.

### Flow Cytometry

In order to stop the secretion of YKL-40 (enrichment of antigen inside the cells), LOX and U87 cells were incubated with 3 µg/ml Brefeldin A (eBioscience) in culture medium for 6 h at 37°C, 5% CO_2_. Cells were detached with enzyme-free Cell Dissociation Buffer (Invitrogen, Karlsruhe, Germany) at room temperature, fixed by incubation with 2% formaldehyde, 0.5% BSA in PBS and permeabilized with 0.5% saponin (Sigma), 0.5% BSA in PBS. Cells were washed with PBS supplemented with 1% BSA between all steps. Anti-YKL-40 antibody or IgG2b isotype control were used at 1 µg/ml for staining of up to 1×10^6^ permeabilized tumor cells in 100 µl PBS, 1% BSA. After washing, cells were incubated with 1∶200 allophycocyanin-conjugated goat anti-mouse Ig (eBioscience) and subjected to fluorescence assisted flow cytometry on a FACSCalibur (BD, Heidelberg, Germany). Files were analyzed using Win MDI 2.9 software.

### Histochemistry

Immunohistochemistry using sections of paraffin-imbedded tumors was carried out as previously described [30]. Briefly, after antigen retrieval (microwave, citrate buffer, ph = 6), mouse anti-YKL-40 or corresponding isotype control (see above) was used as first antibody, respectively, followed by incubation with biotinylated rabbit anti-mouse (Dako, Glostrup, Denmark) and visualization using the streptavidin-alkaline-phosphatase based Vectastain ABC kit (Vector, Burlingame, CA, USA). Slides were scanned by a Mirax microscope (Zeiss, Jena Germany) and the Mirax Viewer (Zeiss) software was used to take images. Other antibodies used for immunohistochemistry (with corresponding biotinylated secondary antibodies (all Dako)) were: Anti murine EDG-1 (S1P1) (Santa Cruz, Heidelberg, Germany), anti-murine CD45 (clone 30-F11, BD) and anti-human Ki-67 (clone MIB-1, Dako). Images of four randomly chosen fields of vision (not containing necrotic areas) were taken with the viewer software. Human Ki-67 or murine CD45 positive cells were counted and murine S1P1 positive areas quantified using the ImageJ software (Wayne Rasband, NIH, USA). Positive cells or percent positive area per field of vision were calculated for each tumor.

Histochemical Masson-Goldner staining of paraffin-imbedded tumors was performed as described [33].

### MRI Measurements

The animals were anesthetized for approximately 45 minutes by intraperitoneal injection of a weight-adapted dose (10 µl/g bodyweight) of a mixture of 1.2 ml Ketamin (Gräub AG, Bern, CH), 0.8 ml Rompun (Bayer AG, Leverkusen, Germany) and 8 ml saline (Invitrogen). MRI was performed on a clinical 3.0 T MRI scanner (Intera, Philips Medical Systems, Best, The Netherlands). The scanner was equipped with a conventional body transmit coil and gradient system allowing a maximal amplitude of 30 mT m^-1^ and a slew rate of 50 mT^-1 ^s^-1^. For signal reception a dedicated four-element mouse coil (Philips Research Laboratories, Hamburg, Germany) with an inner diameter of 2.5 cm was used. The MR sequence protocol consisted of a T1 weighted localizer sequence in three orthogonal planes, an additional T2 weighted localizer in sagittal orientation followed by a high-resolution T1 weighted 2D turbo spin-echo sequence (TSE) in coronar orientation, a fat saturated T2 weighted 2D TSE in coronar and sagittal orientation and a T2* weighted 2D gradient-echo sequence. Image parameters of the whole body coronar T1 TSE sequence were as follows: time of repetition (TR)/time of echo (TE)/flip angle (FA) = 1275 ms/33 ms/90°; field-of-view (FOV) = 100×35 mm^2^; matrix = 464×464; slice thickness (ST) = 1 mm, number of slices (NS) = 14; number of acquisitions (NA) = 3; spatial resolution = 0.22×0.22×1 mm; acquisition time = 5 minutes 18 seconds. Image parameters of the fat saturated whole body coronar and sagittal T2 TSE sequence were as follows: TR/TE/FA = 2361 ms/90 ms/90°; FOV = 100×35 mm^2^; matrix = 448×448; ST = 1 mm, NS = 14; NA = 3; spatial resolution = 0.22×0.22×1 mm; acquisition time = 3 minutes 37 seconds. Image parameters of the whole body T2* gradient-echo sequence sequence were as follows: TR/TE/FA = 252 ms/7 ms/15°; FOV = 100×35 mm^2^; matrix = 464×464; ST = 1 mm, NS = 14; NA = 5; spatial resolution = 0.22×0.22×1 mm; acquisition time = 3 minutes 27 seconds. Tumor size was determined by measuring the largest dimensions of the tumor in the coronar and sagittal plane. Tumor volume was calculated using the following formula assuming an ellipsoid volume: V = 4/3 π abc.

### Tube Formation Assay

Tube formation assays were performed with HUVECs in µ-Slides Angiogenesis (Ibidi, Munich, Germany) using U87-conditioned ECM (see **2.2**.), supplemented with 0, 5 or 10 µg/ml anti-YKL-40 antibody. 10^4^ cells were seeded in 50 µl/µ-Slide well (each condition in triplets) and tube formation was evaluated after 12 h using a standard light microscope with camera and the angiogenesis PlugIn of ImageJ software (Wayne Rasband, NIH, USA).

### Statistical Analysis

Statistical Analysis was done using GraphPad Prism 5 (GraphPad, La Jolla, CA, USA). Tests used are given for each value of P in the Results section.

## Results

### LOX Melanoma Cells Express YKL-40 *in vitro*

**Figure 1:**
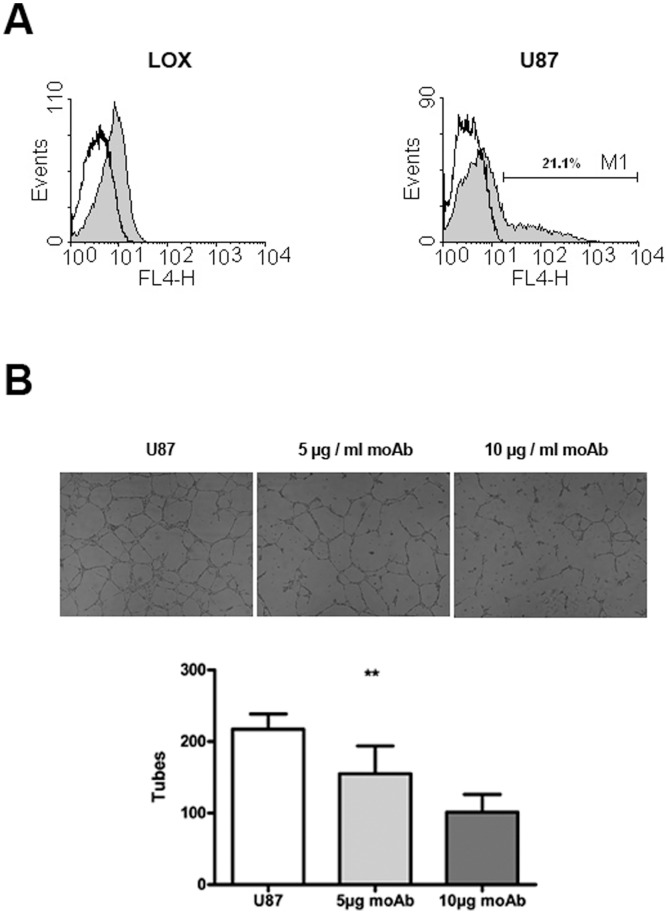
LOX melanoma cells express YKL-40 *in vitro*: FACS anaylsis of *in vitro* expression of YKL-40 by LOX and U87 cells. There is a subpopulation of 21.1% with strong YKL-40 expression (M1) in the U87 cells (**A**). Anti-YKL-40 significantly reduces tube formation: Tube formation of HUVECs in U87 conditioned medium after 12 h. Medium was additionally supplemented with: Nothing (U87), 5 µg/ml anti-YKL-40 mononclonal antibody (5 µg/ml moAb) or 10 µg/ml anti-YKL-40 (10 µg/ml moAb). Representative photographs of wells with tube formation (upper panel) and column bar graph (means with SD, lower panel). The differences were significantly different (** : P = 0.0086, 1 way ANOVA) (**B**).

The human melanoma cell line LOX and the human glioblastoma cell line U87 (as control) were stained intracellularly for YKL-40 protein expression. For the LOX cells there was a moderate but distinct shift compared with the isotype control, the homogeneous peak indicating a weak to moderate YKL-40 expression in all cells (**Figure 1A**). Without addition of Brefeldin A prior to staining, no intracellular YKL-40 could be detected in LOX cells. Most of the U87 cells had a weak YKL-40 expression (small shift for most of the cells). However, a subpopulation of 21% of the cells displayed medium to strong YKL-40 expression (**Figure 1B**). GeneChip (Affimetrix, Santa Clara, CA, USA) analysis previously demonstrated that PaCa 5061 cells do not express YKL-40 [29], data not shown.

Although LOX and U87 cells expressed YKL-40 *in vitro*, the treatment with various concentrations of anti-YKL-40 antibody did not increase or decrease the proliferation rate of either cell line. Also, additional 1 µg/ml of recombinant YKL-40 did not change the proliferation rate of the cells alone or under the influence of anti-YKL-40 (data not shown).

### Anti-YKL-40 Inhibits Tube Formation

In tube formation assays using HUVECs, anti-YKL-40 antibody significantly reduced tube formation by HUVECs in U87-conditioned medium (Figure 1C): In U87-conditioned medium without anti-YKL-40 a mean of 217.3+/−12.35 tubes was observed, compared with a mean of 155+/−22.5 (5 µg anti-YKL-40) and 101.3+/−26.73 (10 µg anti-YKL-40) tubes. These differences were significant (P = 0.0086, 1 way ANOVA).

### Anti-YKL-40 Monoclonal Antibody Enhances Growth of LOX Melanoma Tumors *in vivo*

During all of the studies described below, no animal reached termination criteria other than primary tumor size during the experiments.

#### Study A

**Figure 2:**
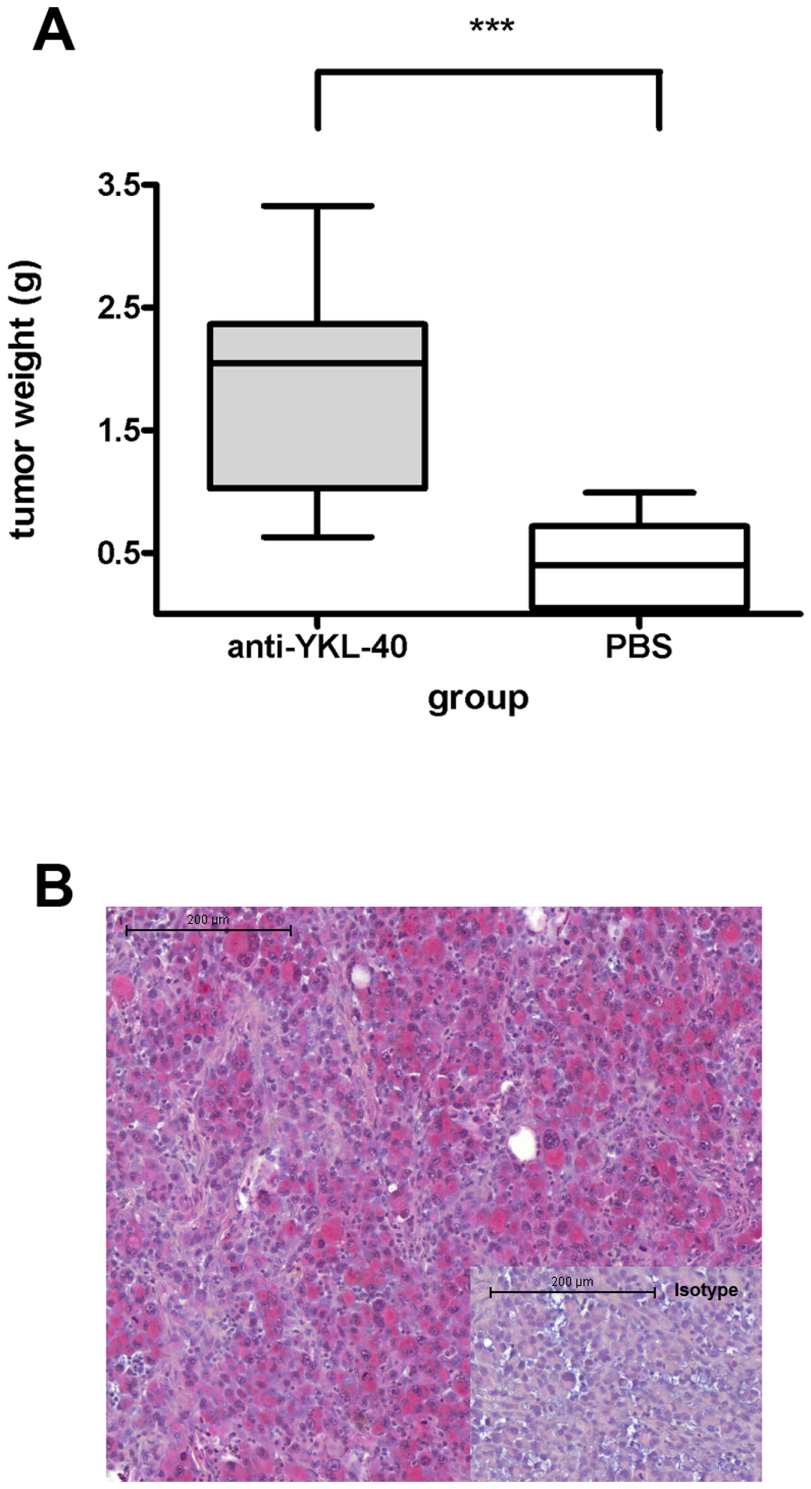
Anti-YKL-40 treatment increases tumor size: Mean tumor weight 18 days after injection of 10^6^ LOX cells in Balb/c scid mice treated with and anti-YKL-40 monoclonal antibody (anti-YKL-40) versus controls (PBS). ***: P<0.0001, unpaired, two tailed t-test (**A**). LOX melanoma cell express YKL-40 in xenograft tumors: Immunohistochemical staining of a LOX tumor grown in a Balb/c scid mouse for 18 days. Staining with an anti-YKL-40 antibody; red color corresponds to positive YKL-40 expression in the melanoma cells. Isotype: Staining with IgG2b isotype control (**B**).

Two days after the injection 1×10^6^ melanoma cells per animal, the mice received the first injection of anti-YKL-40 monoclonal antibody or PBS as control, respectively. Two days after the first antibody injection, tumor growth could be detected manually under the skin of the antibody treated animals whereas the controls showed no (manually detectable) tumor growth at that time. Two mice of the control group died for unknown reasons after their first PBS injection. After 18 days 4 tumors of the anti-YKL-40 antibody treated group exceeded 10% of the animals’ weight and the experiment was therefore ended. Mean tumor weight of the anti-YKL-40 treated animals was more than four times that of the controls (1.817 g ±0.171 g versus 0.418 g ±0.077 g; P<0.0001, unpaired, two tailed t-test; **Figure 2A**). Immunohistochemical staining showed that all tumors displayed YKL-40 protein expression (**Figure 2B**) with no visible difference in expression levels between the groups. In both groups, no metastasis could be detected in serial lung sections.

#### Study B

For the PaCa 5061 cells no enhancement of tumor growth was seen. Median survival was 51 days for the anti-YKL-40 and 61 days for the isotype treated animals. Survival curves were not significantly different (P = 0.4802, Log Rank (Mantel Cox) test and P = 0.6466, Gehan-Breslow-Wilcoxon test, data not shown).

### Following Tumor Growth by MRI after Treatment with anti- YKL-40 Antibody

#### Study C

**Figure 3:**
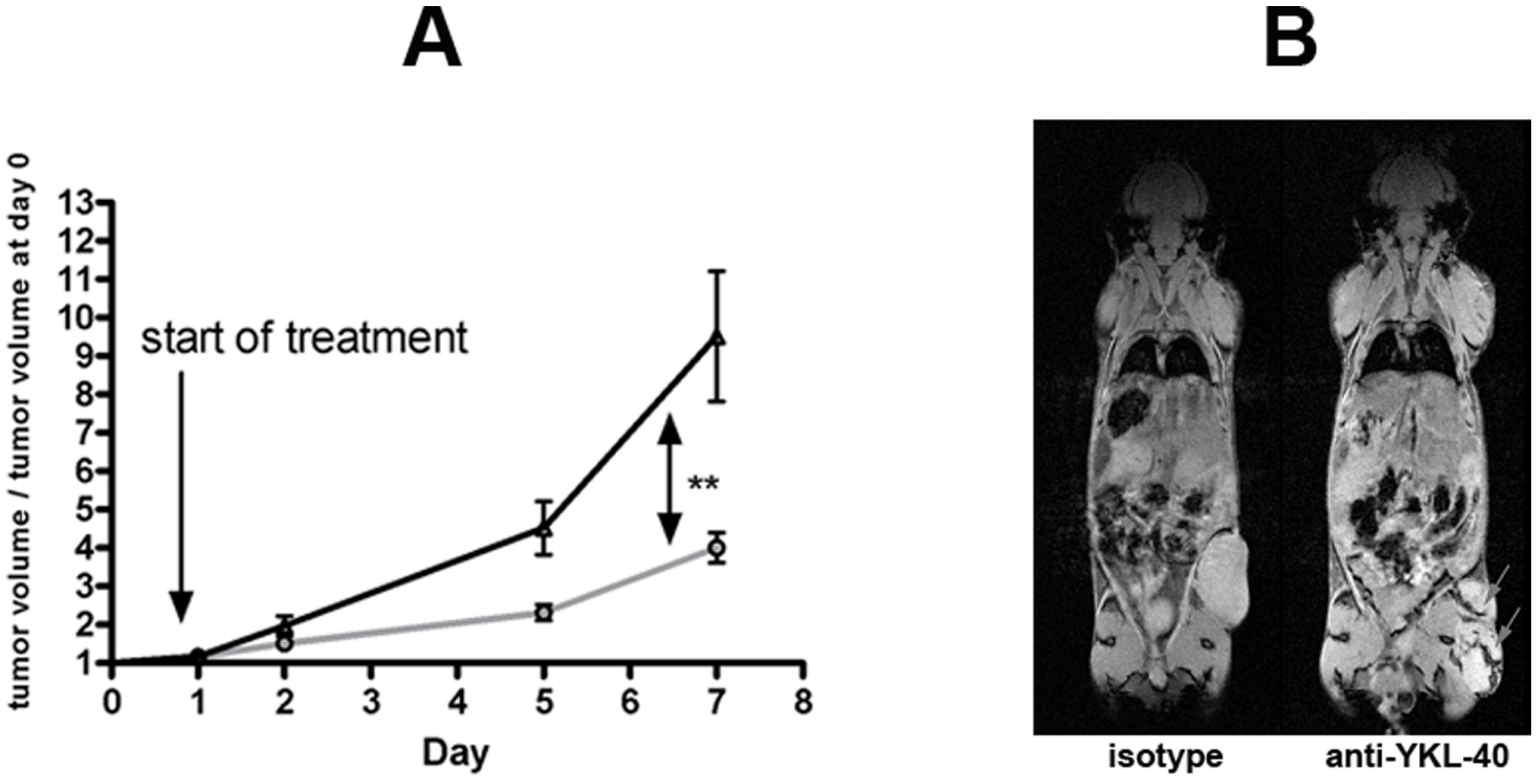
Tumor growth as observed by MRI after injection of 10^6^ LOX melanoma cells during treatment with anti-YKL-40 monoclonal antibody or isotype control. For each animal, tumor volume calculated on basis of MRI data at each time point was divided by the tumor volume at day 0. **Black curve:** treatment with anti-YKL-40 antibody; **grey curve:** treatment with isotype control; **: P = 0.0011, two way ANOVA (**A**). T2* weighted MRI of two Balb/c scid mice with LOX tumors treated with isotype or anti-YKL-40 monoclonal antibody seven days after start of treatment. Observed hemorrhage is indicated by arrows (**B**).

**Figure 4:**
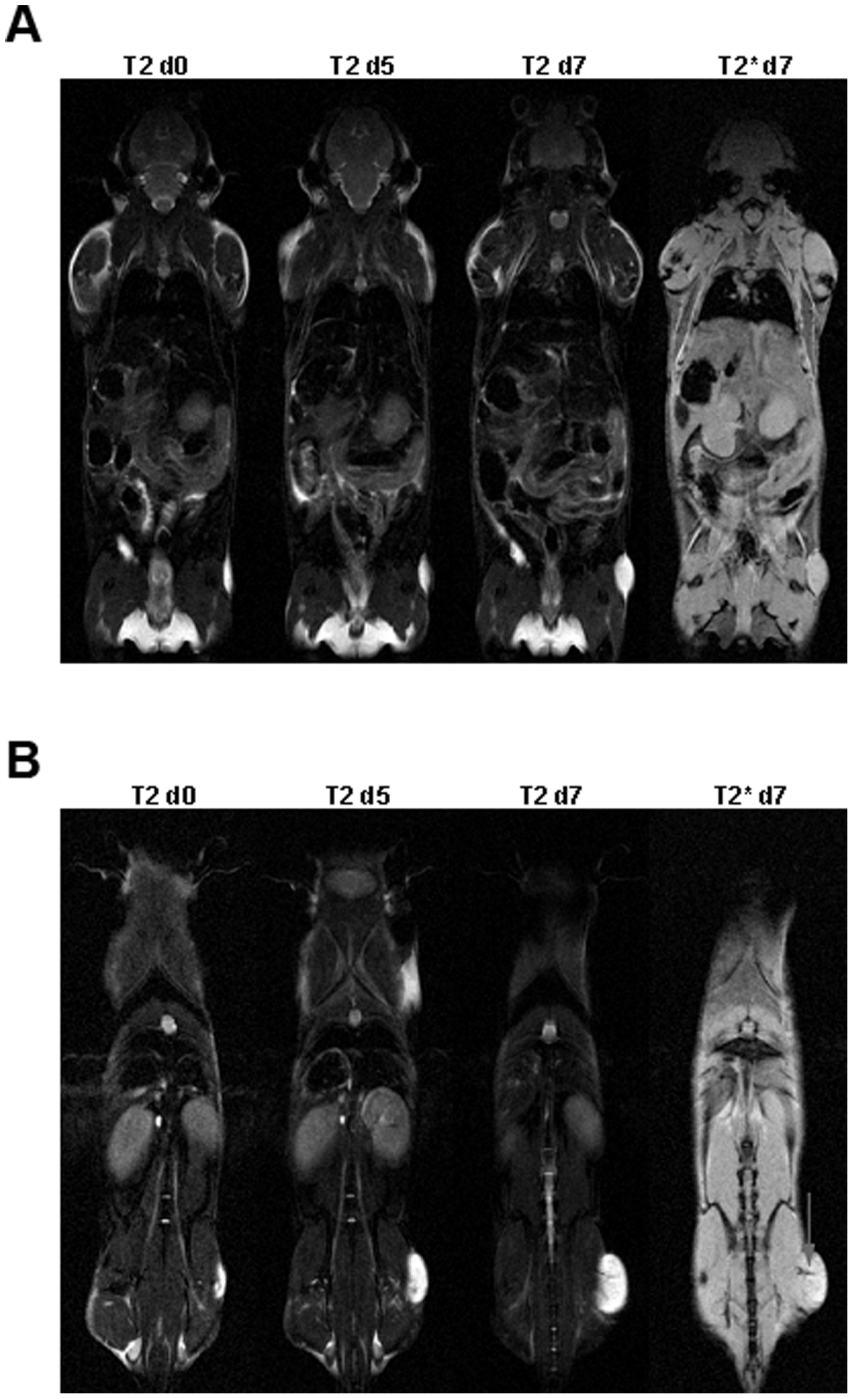
Development of tumor volume with and without anti-YKL-40 treatment. T2 and T2* weighted MRI of two Balb/c scid mice with LOX tumors treated with isotype (**A**) or anti-YKL-40 monoclonal antibody (**B**) before injection of antibody (d0), five days (d5) and seven days (d7) after start of treatment. Observed hemorrhage is indicated by arrow.

10 mice were injected with 10^6^ LOX melanoma cells and after one week 5 mice were treated with anti-YKL-40 antibody and (to exclude unspecific effects of the IgG2b antibody) 5 mice with isotype control. Tumor volume was documented by MRI at 4 time points (6 hours, day 2, day 5 and day 7). Tumor volume increased significantly in the anti-YKL-40 treated mice (**Figure 3A**; P = 0.0011, two way ANOVA). Tumor volumes for each time point and animal were divided by the respective tumor volume of the first MRI measurement, thus both curves start at a value of 1 on day 0. Tumor growth in the anti-YKL-40 treated mice was boosted immediately after the first injection of antibody (**Figure 3A**). At the end of the experiment at day 7, the mean tumor volume of the anti-YKL-40 treated mice had increased 10 fold compared to a 4 fold increase in the mice treated with isotype antibody. The MRI measurements at day 5 and 7 showed signs of bleeding in and around the tumor in three and four mice treated with anti-YKL-40 antibody (**Figure 3B**). MRI scans pretreatment and at day 5 and 7 for one mouse of the control group and one of the anti-YKL-40 treated group are illustrated in **Figure 4**.

### Treatment with anti-YKL-40 Leads to Bleeding at the Tumor Site

#### Study D

**Figure 5:**
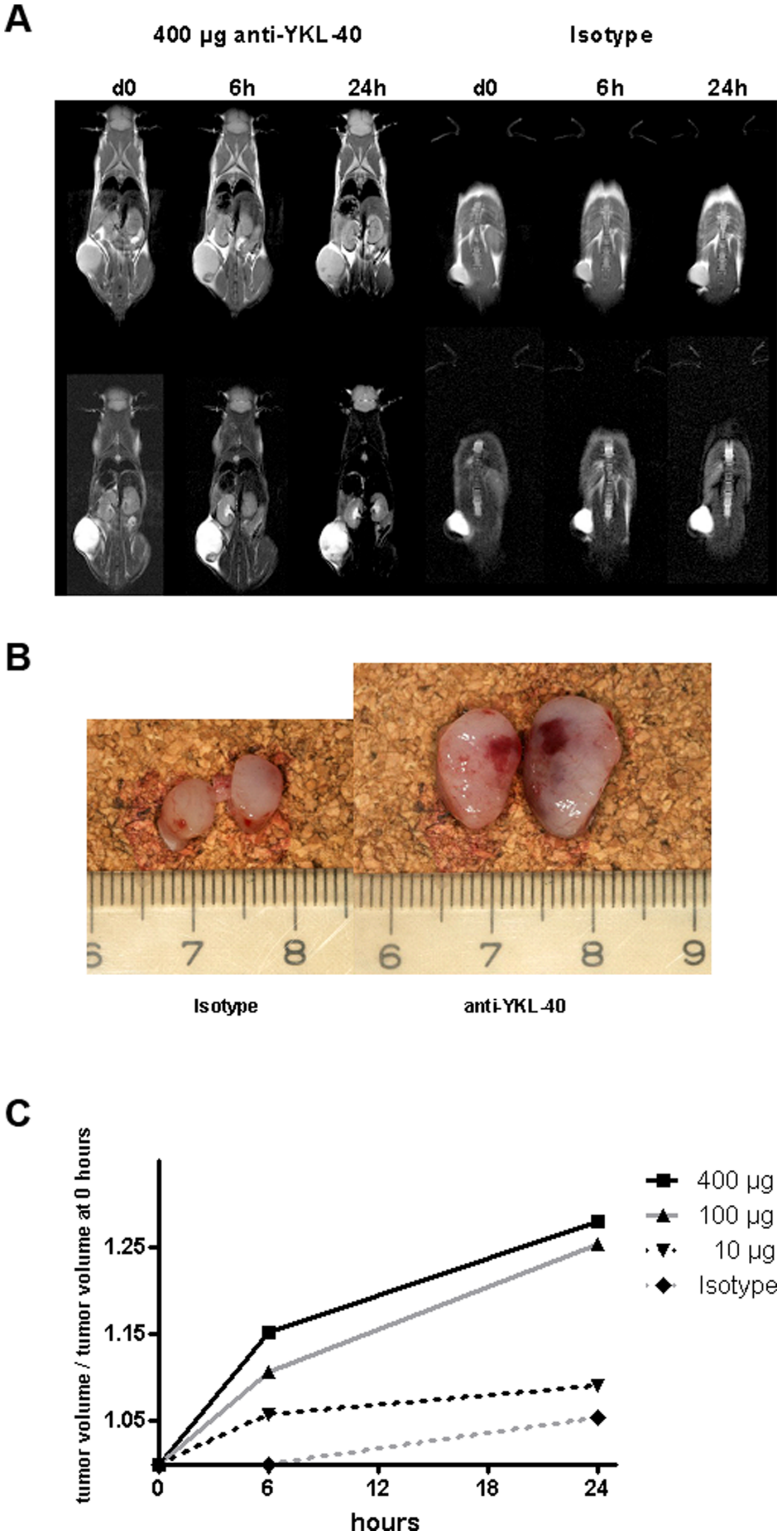
Rapid effect of anti-YKL-40 treatment: T1 and T2 weighted MRI of two Balb/c scid mice with LOX tumors treated with anti-YKL-40 monoclonal antibody or isotype control before injection of antibody (d0), six hours (6 h) and 24 hours (24 h) after antibody injection. Bleeding can be seen as dark shades in the tumor (**A**). Two dissected tumors (each cut in half) 24 hours after i.v. treatment with anti-YKL-40 antibody or isotype treatment (**B**).

To verify early increase in tumor volume and bleeding, four animals received an injection of 10^6^ LOX melanoma cells in the flank. After one week of tumor growth the mice received intravenous injections of 10, 100 or 400 µg of anti-YKL-40 antibody or 400 µg of isotype control, respectively. MR imagings were taken 12 h before injections and 6 h and 24 h after. Bleeding at the tumor site was detected after 6 h and 24 h in animals treated with 100 and 400 µg of anti-YKL-40, but not for 10 µg and the isotype control (**Figure 5A**). Immediately after the second MRI examination (24 h after injection) the mice were sacrificed, tumors taken out and cut in half. Coagulated and fresh bleeding was seen in the tumors from the mice treated with 100 and 400 µg anti-YKL-40 antibody (**Figure 5B**). The tumor volumes were calculated from MRI data and a dose dependent effect on tumor size for anti-YKL-40 could be observed (probably due to the low animal number, however, not statistically significant: P = 0.199, two way ANOVA; **Figure 5C**).

### Anti-YKL-40 Treatment Leads to Increased Vessel Formation in the Tumors

**Figure 6:**
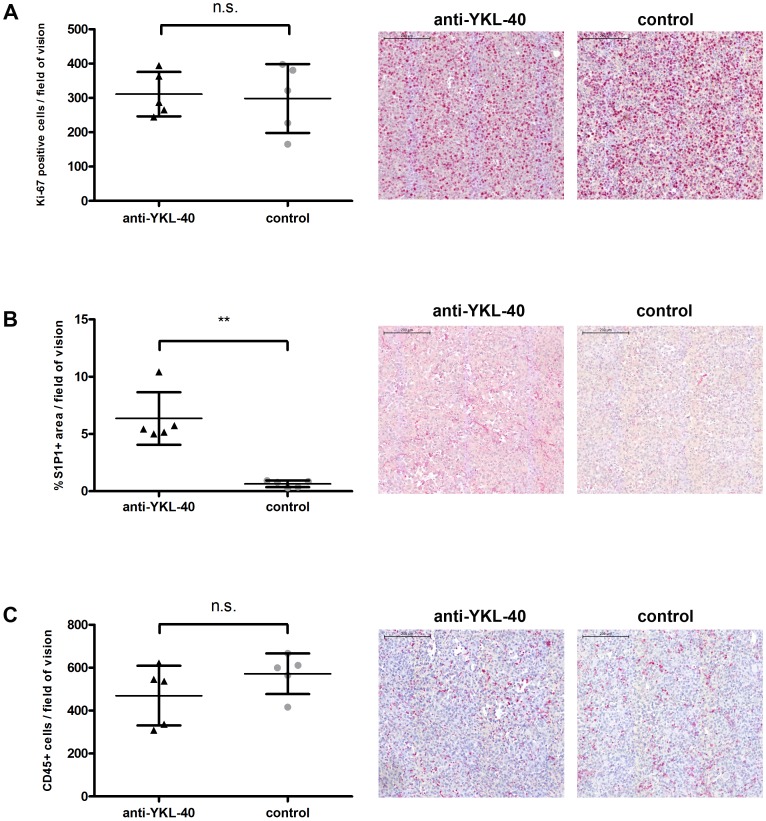
No significant difference in proliferation was observed between anti-YKL-40 treated and control tumors (N = 5, each). Given is the number of human Ki-67 positive cells per field of vision as determined by immunohistochemistry. Representative tumor stainings to the right (**A**). Significant difference in vessel formation between anti-YKL-40 treated and control tumors (N = 5, each). Given is the percentage of murine S1P1 positive area per field of vision as determined by immunohistochemistry. Representative tumor stainings to the right (**B**). No significant difference in leukocyte infiltration was observed between anti-YKL-40 treated and control tumors (N = 5, each). Given is the number of murine CD45 positive cells per field of vision as determined by immunohistochemistry. Representative tumor stainings to the right (**C**).

**Figure 7:**
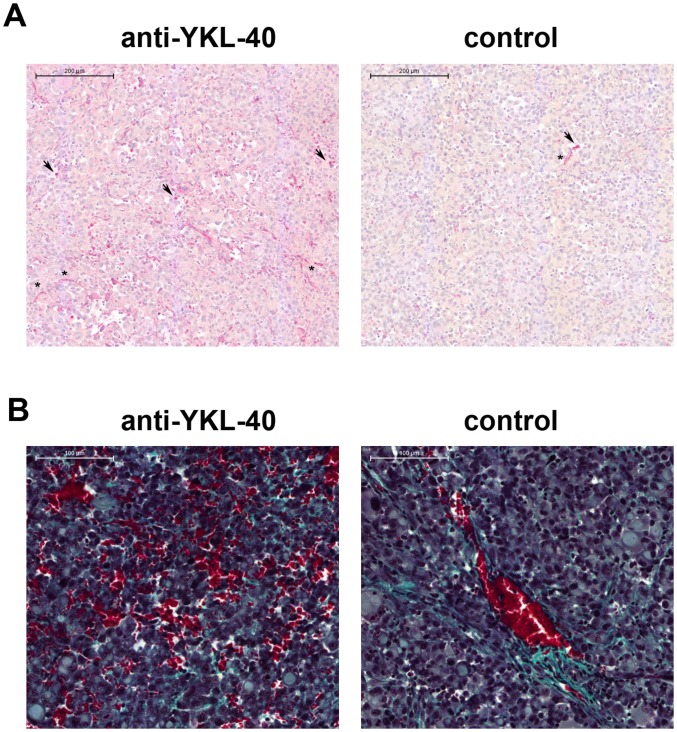
Vessels and single S1P1 positive cells in the xenograft tumors: Immunohistochemical staining of anti-YKL-40 treated and control tumors for murine S1P1 (as in Figure 6B, right panel). Asterisks: vessels; arrows: Single S1P1 positive cells (**A**). Intratumoral bleeding leaves tumor tissue mostly intact: Histochemical Masson-Goldner staining of anti-YKL-40 treated and control tumors. nuclei: brown/black; connective tissue: green; erythrocytes: red (**B**).

To gain more insight into the mechanisms of increased tumor growth and bleeding, proliferation (immunohistochemical staining for human Ki-67, **Figure 6A**) and vessel formation (staining for murine S1P1, **Figure 6B**) were quantified in anti-YKL-40 treated and control tumors. No significant difference in proliferation (Ki-67) was observed, however, formation of new vessels (S1P1 positive) increased significantly in the anti-YKL-40 treated tumors. In addition to clearly recognizable new vessels, a number of single or small groups of cells positive for S1P1 could be observed. To determine if these cells were (murine) leukocytes, the tumors were stained for murine CD45 expression (**Figure 7**): No significant difference in leukocyte infiltration (CD45 expression) between anti-YKL-40 treated tumors and controls could be observed.

Masson-Goldner staining clearly showed areas with damaged vessels (after anti-YKL-40 treatment). Apart from the obviously damaged vessels, tumor tissue structure in these areas remained mostly intact (**Figure 7B**). This finding corresponds with the results from MRI analyses, where especially the T2* weighted 2D gradient-echo sequences showed that not only the smaller control tumors, but also the bigger anti-YKL-40 treated tumors consist of solid tumor tissue (**Figures 3B** **,** **4**). Tumors from both groups were not cystic (consisted of solid tissue) and did not contain extended necrotic areas (**Figure 5B**).

## Discussion

The aim of our study was to analyze the effect of targeting YKL-40 secreted by human melanoma cells with a monoclonal antibody against YKL-40 in a xenograft mouse model. It had been demonstrated that YKL-40 promoted tumor growth in a xenograft experiment [16] and that an anti-YKL-40 antibody significantly reduced tumor growth in a human glioblastoma xenograft model in mice. We therefore expected to see inhibition of tumor growth in this xenograft model of human melanoma, especially as the antibody used in this study lead to a decrease in tube formation (**Figure 1B**) – an effect described for another monoclonal anti-YKL-40 antibody [28]. Surprisingly, no inhibition of tumor growth was found in mice treated with the anti-YKL-40 monoclonal antibody. Instead there was a strong increase in tumor volume comparable with that observed after overexpression of YKL-40 in an otherwise non-YKL-40-secreting cell line [16]. Since there is no forced overexpression of YKL-40 due to genetic manipulation of the tumor cells in the present study and immunohistochemical staining for human YKL-40 showed no visible difference in expression between the tumors of the anti-YKL-40 treated and the control group, the observed boost of tumor volume is probably due to a drastic increase in the effect of YKL-40. Although the anti-YKL-40 antibody caused a decrease in tube formation *in vitro*, the *in vivo* results showed a significant increase in formation of new vessels in the anti-YKL-40 treated tumors (**Figure 6B**). We interpret the single or small groups of S1P1 positive cells found in the anti-YKL-40 treated tumors (**Figure 7A**) which obviously are not leukocytes (**Figure 6C**), as differentiating endothelial cells starting to form new vessels. These observations described above offer a good explanation for the drastic increase in tumor growth.

The anti-YKL-40 monoclonal antibody had no effect on the proliferation rate of LOX melanoma cells *in vitro* (which has also been described for colon and breast cancer cells [16]) and *in vivo* (**Figure 6A**), making a direct effect on the tumor cells *in vivo* unlikely. YKL-40 protein expression is most often found *in vivo* in cancer cells using immunohistochemical studies, whereas very few cancer cell lines (like U87 and MG63 cells) produce YKL-40 *in vitro* (JS Johansen, personal observations). The anti-YKL-40 antibody used in the present study has no affinity to murine YKL-40 (JS Johansen, personal observations) and the YKL-40 possibly produced by mouse stroma cells was therefore not targeted by this antibody. For PaCa 5061 cells, no enhanced tumor growth could be observed in scid mice with anti-YKL-40 treatment. As this cell line does not express YKL-40, this indicates that the observed effect for the LOX tumors was indeed human YKL-40 (secreted by the melanoma cells) specific.

Most striking, however, is the difference between our results and that of the glioblastoma xenograft [28]. The authors of this study observed a 40% decrease in (cell line U87) tumor growth in 5 scid/beige mice treated with an anti-YKL-40 monclonal antibody (mAY) compared with 5 control scid/beige mice whereas we observed a 400% increase in tumor volume in 20 scid mice. One major difference is the mouse strain: The scid/beige mice used in the glioblastoma study are NK-cell deficient whereas the scid mice of our study have functional NK-cells. Today, it is still unclear how or even if YKL-40 acts on NK-cells. The second major difference is the fact that the monoclonal antibodies used in the two studies were not the same. If different epitopes (or different isotypes - unfortunately we were unable to find any reference to the exact isotype of mAY) were the cause for the drastically different effects discussed above, this would be highly interesting as binding two different epitopes of the same molecule would cause more or less opposite effects.

It has been demonstrated that YKL-40 promotes tumor angiogenesis via initiating a coordination of syndecan-1 and integrin α_v_β_3_ and a following intracellular activation cascade in the membrane of endothelial cells [16], [17]. Our monoclonal antibody might increase this coordination by some kind of cross-linking effect – at least it lead to a significant increase in vessel formation in the melanoma xenograft. Since it is unknown where YKL-40 binds to initiate syndecan-1 and integrin interaction, this remains speculation at present. It is also plausible that dimerization of YKL-40, which is a lectin [6], enhances its affinity and avidity for whatever carbohydrate ligand it binds to *in vivo*. However, the tube formation assay (the only available *in vitro* test for the blocking function of an anti-YKL-40 antibody) yielded more or less the same result for the antibody used in this study as for mAY [28]. It is thus rather difficult to see a functional difference between the two anti-YKL-40 antibodies. The intra-tumoral hemorrhage observed by MRI, especially on the T2* weighted gradient-echo sequence, shortly after antibody treatment is an additional indication that the observed effect is due to an interaction of YKL-40 and anti-YKL-40 with the endothelium of vessels surrounding the tumor. The short time from anti-YKL-40 antibody injection to massive bleeding at the tumor site is also remarkable.

A third major difference is of course the tumor entity: Glioblastoma versus melanoma. However, as YKL-40 produced by the tumor cells is thought to act on mouse cells/tissues rather than on the (human) tumor cells themselves, [16], [17], [28] plus there is no difference in proliferation of LOX cells under influence of anti-YKL-40, it is not easy to explain how the different entities might cause either an in- or decrease in tumor volume.

In conclusion, in a xenograft model of human melanoma in scid mice, we found that monotherapy with an antibody targeting YKL-40 rapidly enhances melanoma tumor size at least in part by increasing the formation of new tumor vessels. YKL-40 might still be an interesting target for anti-tumor therapy. However, monotherapy with anti-YKL-40 antibody might be a problematic approach at least in certain tumor entities. Therefore, many important questions remain to be solved concerning the mode of action of YKL-40 which remains an enigmatic molecule.
